# Sphingosine kinase 2 supports the development of BCR/ABL-independent acute lymphoblastic leukemia in mice

**DOI:** 10.1186/s40364-018-0120-4

**Published:** 2018-02-05

**Authors:** Vicki Xie, Daochen Tong, Craig T. Wallington-Beddoe, Ken F. Bradstock, Linda J. Bendall

**Affiliations:** 1 Centre for Cancer Research, The Westmead Institute for Medical Research, The University of Sydney, Sydney, Australia; 2 Haematology Department, Westmead Hospital, Westmead, NSW Australia; 3 Centre for Cancer Biology, University of South Australia and SA Pathology, Adelaide, Australia; 4 College of Medicine and Public Health, Flinders University, Adelaide, Australia; 5 School of Medicine, University of Adelaide, Adelaide, Australia

**Keywords:** Acute lymphoblastic leukemia, Sphingosine kinase 2, Mouse models

## Abstract

**Background:**

Sphingosine kinase (SphK) 2 has been implicated in the development of a range of cancers and inhibitors of this enzyme are currently in clinical trial. We have previously demonstrated a role for SphK2 in the development of acute lymphoblastic leukemia (ALL).

**Methods:**

In this and our previous study we use mouse models: in the previous study the disease was driven by the proto-oncogene BCR/ABL1, while in this study cancer risk was elevated by deletion of the tumor suppressor ARF.

**Results:**

Mice lacking ARF and SphK2 had a significantly reduced incidence of ALL compared mice with wild type SphK2.

**Conclusions:**

These results show that the role of SphK2 in ALL development is not limited to BCR/ABL1 driven disease extending the potential use of inhibitors of this enzyme to ALL patients whose disease have driver mutations other than BCR/ABL1.

**Electronic supplementary material:**

The online version of this article (10.1186/s40364-018-0120-4) contains supplementary material, which is available to authorized users.

## Background

There are two forms of sphingosine kinase (SphK), SphK1 and SphK2. SphK1 has an established role in malignant biology with overexpression being associated with poor survival in patients with solid tumors [1–10] and resistance to therapy [11–14]. Furthermore, inhibitors of SphK1 have demonstrated preclinical activity in acute myeloid leukemia (AML) [15, 16]. The role of SphK2 has been more controversial but it is increasingly being shown to play a role in malignant disease and has been associated with poor patient outcome [17]. Knockdown of SphK2 expression increases the sensitivity of cancer cells to chemotherapy [18–20], while chemical inhibition can reduce cancer cell growth in vitro [21–28] and in pre-clinical animal models [21, 24, 26]. SphK2 inhibitors are now in phase II clinical trials for a number of cancers including B cell malignancies, following successful completion of phase I studies [29]. We have recently shown that chemical inhibition of SphK2 can reduce acute lymphoblastic leukemia (ALL) cell growth, induce cell death in vitro and extend the survival of mice bearing human ALL xenografts. Furthermore, cells lacking SphK2 had a reduced capacity to induce ALL driven by the BCR/ABL1 fusion gene in WT mice, while SphK2 inhibition synergized with imatinib treatment of BCR/ABL1+ ALL in vitro and in vivo [30].

Mice deficient in the tumor suppressor gene ARF are prone to malignancies, with undifferentiated sarcomas predominating (~ 38%), followed by lymphomas (~ 23%), carcinomas (~ 15%) and neurological tumors (~ 10%), with a latency of around 266 days [31]. Genetic loss of material at the 9p21 locus, which includes ARF, is common in ALL, being reported in up to 45% of B lineage disease [32–34], making this a biologically relevant model. The development of tumors in these mice appears to be dependent on the aquisition of additional genetic changes as treatment with radiation or the mutagen DMBA significantly reduces latency. Here we show that blockade of T and B cell maturation by crossing ARF deficient mice onto a Rag1^−/−^ background [35] resulted in an incidence of ALL of over 60%. Further crossing of these mice onto SphK2 deficient animals [36] permitted the examination of the role of SphK2 in the development of ALL, demonstrating a significant reduction in disease incidence.

## Methods

### Development of mouse model

Mice lacking the p19ARF product of the INK4a/ARF locus (ARF^−/−^) develop malignancies at a high penetrance with 80% of animals dying within the first year of life [31]. To facilitate breeding we used mice where the ARF gene had been floxed (ARF^fl/fl^) (B6.129-Cdkn2atm4Cjs/Nci, [37]) obtained from Graham Walker (QIMR, Queensland Australia). In order to produce an ALL model we crossed these mice with those lacking Rag1^tm1Mom^ from The Jackson Laboratory (Bar Harbour, ME, USA) [35]. The resulting Mx1.Cre.ARF^fl/fl^.Rag1^−/−^ (MAR) mice were then crossed onto animals lacking SphK2 (Richard Proia (Bethesda, USA) [36]) to produce Mx1.Cre.ARF^fl/fl^.Rag1^−/−^.SphK2^−/−^ animals (MARS2 mice). The deletion of the ARF gene was undertaken at 6 weeks of age by intraperitoneal injection of 15 mg/kg of PolyI:polyC every second day for a total of 3 doses and confirmed by PCR (Additional file 1: Figure S1). All mice were obtained or were backcrossed onto on a C57Bl6 background. Experimental mice were monitored for up to 400 days. Mice were defined as having ALL when at the time of death the bone marrow and spleen primarily consisted of B220^+^CD19^+^Gr1^−^ cells. Survival was analysed using the Kaplan-Meier method and SPSS Statistics software.

Mice were genotyped by PCR on genomic DNA obtained from ear punches using DirectPCR Lysis Reagent (Ear) (Viagen Biotech, Los Angeles CA) with 0.4 mg/mL proteinase K (Promega, Alexandria, NSW, Australia) (complete lysis solution). Ear punches from mice were incubated in complete lysis solution for 2 h at 56 °C and proteinase K was inactivated for 30 min at 85 °C prior to PCR. Deletion of ARF was detected in genomic DNA obtained from spleen cells recovered from culled mice. PCR reactions were performed using MyTaq DNA polymerase (Bioline, Eveleigh NSW Australia) and specific primers as indicated in Additional file 1: Table S1. The IL-2 PCR was used as a positive DNA control for the Mx1.Cre reaction. The PCR conditions were 95 °C for 1″, then 95 °C for 15″, 58 °C for 15″, 72 °C for 20″ for 35 cycles, 72 °C for 5′. Amplified products were separated on a 2% agarose (Sigma-Aldrich) gel stained with Midori Green Nucleic Acid solution (Bulldog Bio Inc., Portsmouth NH) and visualised using ChemiDoc MP Imaging System (Bio-Rad, Hercules, CA).

### Flow cytometry

Flow cytometry was performed using a FACSCanto 6-colour flow cytometer (BD Biosciences, San Jose CA). The following antibodies were purchased: Sca-1-PE-Cy7, c-Kit-APC, CD43-APC, IgM-PCP.Cy5.5, IgM-Biotin (Australian Biosearch, WangarraWA, Australia); B220-APC.Cy7, B220 PE-Cy5, CD11b-PE, CD11b-FITC, CD19-PE, CD19-APC.Cy7, Gr1-FITC, Streptavidin APC and Lineage Cocktail of biotinylated CD3, Gr-1, Ter119, B220 and CD11b (BD Biosciences, San Jose CA), and Streptavidin Pacific Blue (Thermofisher Scientific, North Ryde, NSW, Australia). Cells were labelled with antibodies as previously described [30].

### Histology and image acquisition

Blood films were prepared and stained with a Romanowsky stain. Tissues were fixed in 10% formalin, embedded, sectioned and stained as previously described [38]. Femurs were decalcified prior to embedding as previously described [38]. Images were obtained using a NanoZoomer Slide Scanner (SDR Scientific, Sydney Australia) or an Olympus BX51 microscope with images captured using a Spot RT slider camera (Diagnostic Instruments, Sterling Heights, MI) and SPOT Advanced software. Composite figures prepared using Adobe Photoshop software.

## Results

### Deletion of ARF in Rag1 deficient mice predisposes to ALL

Mice lacking ARF are known to develop malignancies with an increased incidence [31]. To generate an ALL model we bred Mx1.Cre.ARF^fl/fl^ mice with Rag1^−/−^ mice to generate Mx1.Cre.ARF^fl/fl^.Rag1^−/−^ mice. At 6 weeks of age mice received 3 injections of polyI:polyC to delete the ARF gene producing Mx1.Cre.ARF^−/−^.Rag1^−/−^ (MAR) mice.

**Fig. 1:**
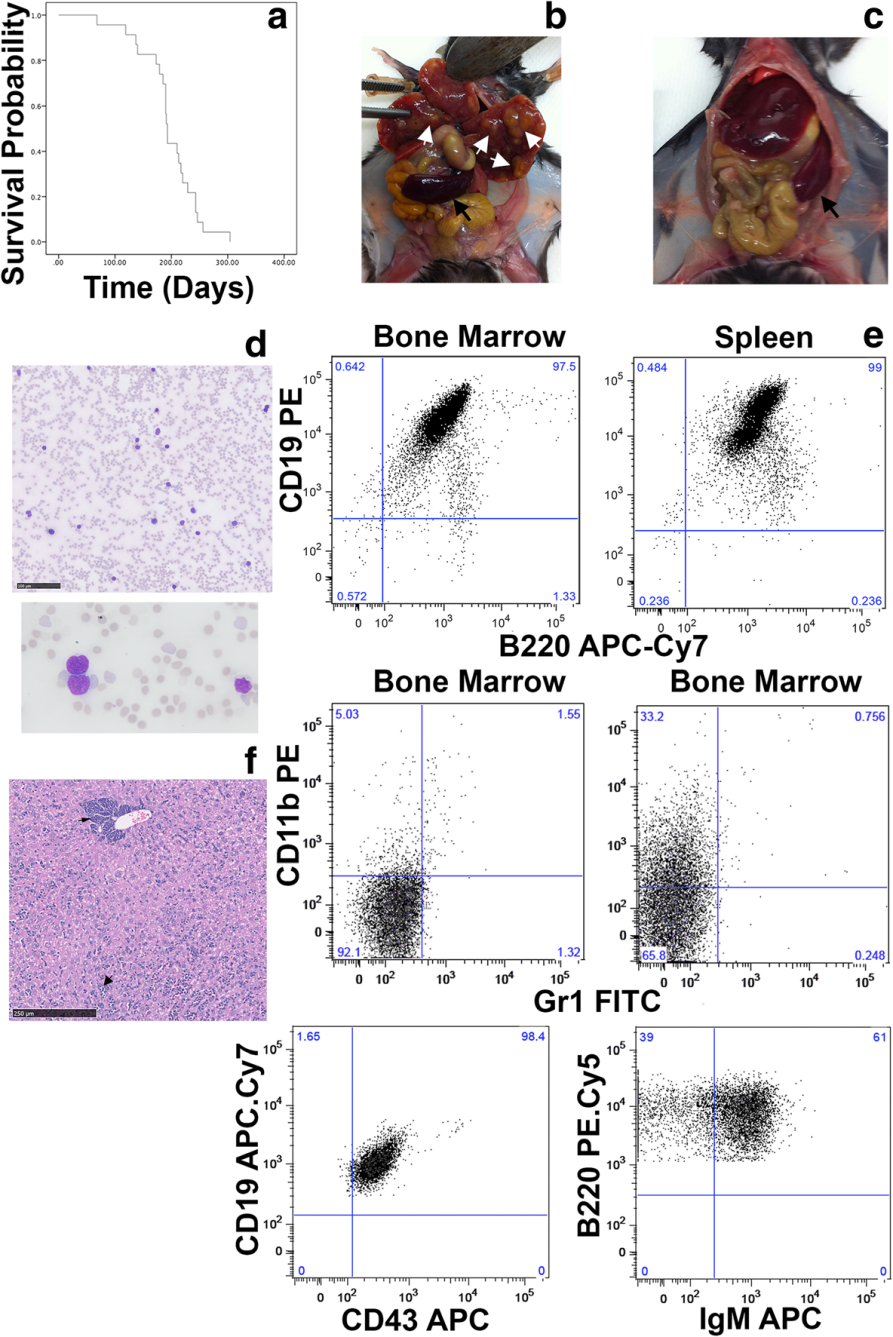
MAR mice develop malignancies with B lineage ALL predominating. **a** Kalpan-Meyer analysis showing the survival of MAR mice. **b** Mouse culled due to disease other than ALL showing tumors in the liver (white arrows) and an enlarged spleen (black arrow). **c** Mouse culled due to ALL showing enlarged spleen (black arrow). **d** Blood film from a mouse with ALL showing circulating lymphoblasts. Image acquired using a slide scanner and size bar represents 100 μm. Lower imaged taken on a Spot camera, original magnification 600×. **e** Flow cytometric analysis of bone marrow and spleen cells from mice culled due to ALL. Upper panels are from the same mouse. Central panels show the lowest and highest CD11b expression detected. Lower panels show typical expression of maturation markers B220, CD19, CD43 and surface IgM. Quadrants were set based on control stained cells from the same animal. **f** Section of liver from a mouse culled due to ALL showing both perivascular (thin arrow) and diffuse (thick arrow) infiltration by ALL cells. The degree of infiltration in this animal was typical. Image acquired using slide scanner and size bar indicates 250 μm

Rag1^−/−^ mice with deleted ARF (MAR mice) survived for up to 304 days (median 193 days) (Fig. 1a). The most common cause of death was B lineage ALL, which occurred in 61% of mice between 119 and 243 days with a median of 192 days. The remaining animals succumbed to a number of causes including other haematological malignancies, with the most common feature of non-ALL deaths being massively enlarged pale livers that sometimes contained defined tumors (Fig. 1b). However the origin of the tumors could not be determined with certainty. Many appeared to be haematological in origin based on morphology but the bone marrows mostly appeared normal (Additional file 1: Figure S2). Flow cytometric analysis of cells recovered from the bone marrow and spleens of these animals was generally uninformative.

Mice that developed ALL were easily identified, demonstrating weight loss, reduced activity and/or impaired use of hind limbs and tail. One displayed hydrocephaly, with fitting. Necropsy findings were consistent with B lineage ALL with enlarged spleens and often enlarged livers, without evidence of tumors and a normal dark red colour (Fig. 1c). Mice with ALL also had elevated WBC for immune-compromised mice (median 15.2, range 2.1-286.5 cells/mL) with significant numbers of lymphoblasts present in blood smears (Fig. 1d). Lymph nodes were rarely involved with only 2 mice having visible nodes on cull and only 1 of those having significant lymphadenopathy (Additional file 1: Figure S3). Cells in the spleen and bone marrow were mostly B220 and CD19 positive (average of 73%, range 56-87 and 86%, range 73-97 respectively), lacking staining for the myeloid marker Gr1 and the T cell marker CD3, however CD11b was detected on cells from some animals (Fig. 1e). Cells from all mice with ALL were positive for immature marker CD43 and most expressed IgM on at least a proportion of the cells (Fig. 1e). The lack of lymph node involvement in the vast majority of animals, near complete replacement of the bone marrow by lymphoblasts as well as the expression of the immature marker CD43 and low expression of IgM indicate a pro- to pre-B classification of these leukemias. Other organs, primarily the liver, were infiltrated with lymphoblasts (Fig. 1f). ALL induced death tended to be earlier compared to non-ALL deaths, with the latter occurring between 68 and 304 days with a median of 229 days, although this was not statistically significant, *p* = 0.06) (Additional file 1: Figure S4). Animals that did not develop ALL mostly presented with solid tumors at a slightly later time point.

### Deletion of SphK2 reduced the incidence of B ALL

**Fig. 2:**
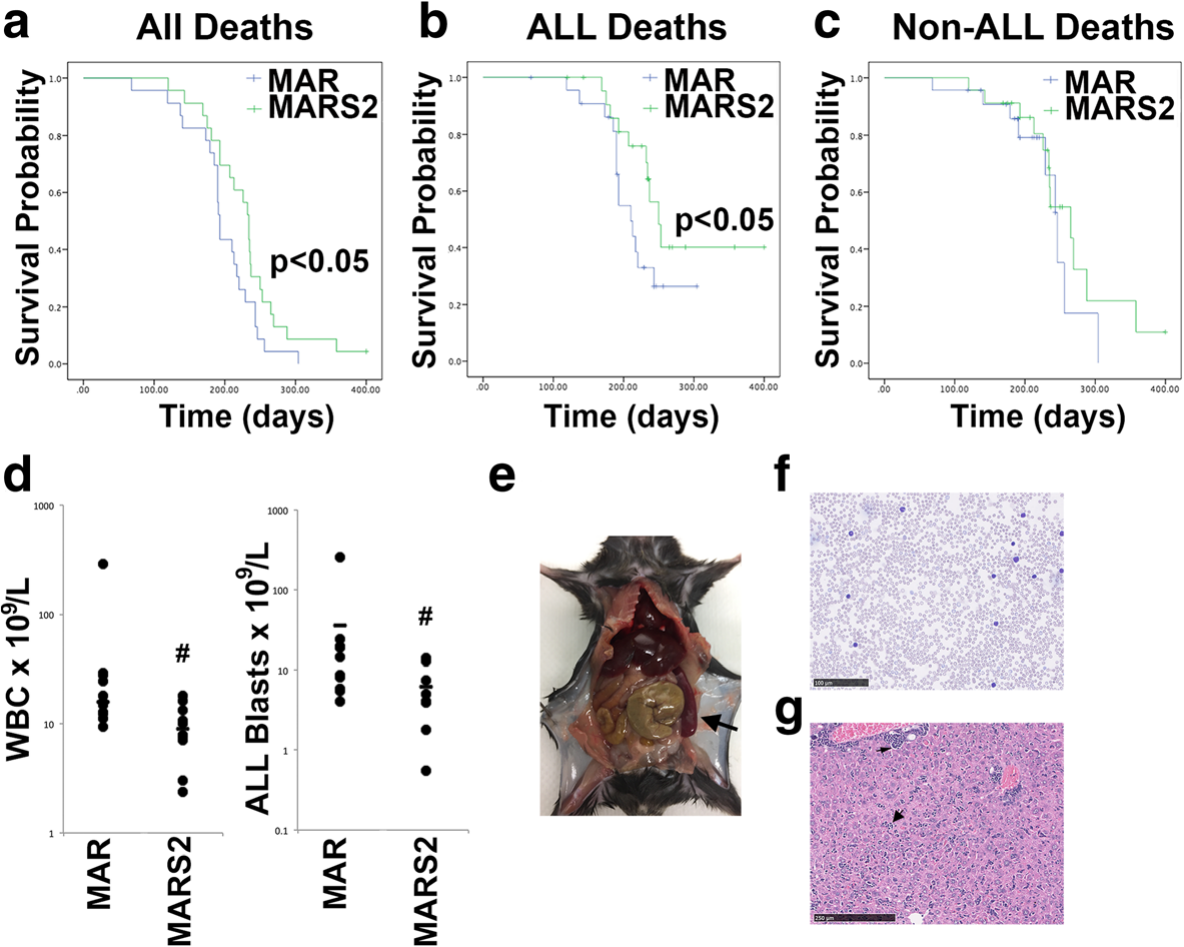
Loss of SphK2 reduces the incidence of B lineage ALL. **a**-**c** Kaplan-Meier plots showing all (**a**) and ALL-induced (**b**) deaths. Deaths due to causes other than ALL are illustrated in (**c**). Total WBC (**d**, left panel) and ALL blast counts (**d**, right panel) at the time of sacrifice are shown. # indicates *p* < 0.05. **e** Mouse culled due to ALL showing enlarged spleen (black arrow). **f** Blood film from a mouse with ALL showing circulating lymphoblasts. Image acquired using a slide scanner and size bar represents 100 μm. **g** Section of liver from a mouse culled due to ALL showing both perivascular (thin arrow) and diffuse (thick arrow) infiltration by ALL cells. The degree of infiltration in this animal was typical. Image acquired using slide scanner and size bar indicates 250 μm

A cohort of mice lacking ARF and Rag1 was also generated using the same methodology on an SphK2^−/−^ background (MARS2 mice). ARF was similarly deleted at 6 weeks of age by 3 injections of polyI:polyC. These mice also largely succumbed to conditions consistent with malignant diseases but compared to MAR mice had significantly increased overall survival with deaths occurring between 120 and > 400 days (one mouse was electively culled disease free at 400 days) with a median of 234 days (*p* < 0.05) (Fig. 2a). Notably there were fewer deaths resulting from ALL in MARS2 animals with only 43% of deaths being due to ALL, resulting in a significant increase in leukemia free survival in MARS2 mice (*p* = 0.044) (Fig. 2b).

The absence of SphK2 did not alter the nature of the ALL that developed, with latency, phenotype and disease dissemination being similar. Death due to ALL was slightly delayed in MARS2 mice (range 169 – 253, median 219.5 days), however this was not significantly different from that of MAR mice (Fig. 2c). Interestingly the WBC in the leukemic MARS2 mice was significantly lower than in the MAR mice, as was the number of circulating blasts (Fig. 2d), however the blast percentage was similar between the two groups. Otherwise the disease was identical in MARS2 and MAR mice, with similar enlargement of spleen and liver and infiltration of other organs (Fig. 2e–g).

## Discussion

Inhibition of sphingosine kinases has recently become of interest for the treatment of a number of conditions including malignant disease [39]. Clinical trials for the SphK2 inhibitor ABC294640, are well under way with phase I studies complete [29] and phase I/II and phase II trials examining hepatocellular carcinoma, Kaposi sarcoma as well as the haematological malignancies multiple myeloma and diffuse large B cell lymphoma ongoing (NCT02229981, NCT02939807 and NCT02757326). These trials have been supported by recent preclinical data from a number of groups [23, 24, 26, 30, 40–44]. The majority of these studies have focussed on solid tumors, however there are reports in haematological malignancies including multiple myeloma [26] and T-ALL [45], and we have previously reported a role for SphK2 in B lineage ALL [30] using a BCR/ABL1-dependent model. In this study, we examined the effects of SphK2 gene deletion on the development of ALL in a model that is not dependent on forced expression of BCR/ABL1 and demonstrated that genetic deletion of SphK2 also inhibits the development of B lineage ALL independent of BCR/ABL1 expression. The similar latency and features of the disease in MAR and MARS2 mice suggests that the principal effect of SphK2 loss was on leukemia initiation rather than rate of disease progression. However, we previously demonstrated that the SphK2 inhibitor ABC294640 impedes disease progression in a xenograft model of Ph^−^ human ALL, suggesting that SphK2 loss/inhibition has some effect on disease progression [30].

The reason why loss of SphK2 decreases the incidence of ALL is not entirely clear. However SphK2 has a well-established role in promoting malignant cell survival [46] making it possible that in the absence of SphK2, cells with newly acquired potentially oncogenic changes are more susceptible to cell death. While precise mechanisms are yet to be determined, one potential explanation relates to CDKN1A expression. CDKN1A is an inhibitor of apoptosis induced in response to DNA damage whose expression is increased by SphK2-mediated effects on histone acetylation [47]. In the absence of SphK2, induction of CDKN1A expression following DNA damage could be reduced increasing the probability of cell death. Another possible mechanism relating loss of SphK2 to the reduced incidence of ALL concerns the localization of SphK2 to the endoplasmic reticulum (ER) membrane and its involvement in sphingolipid metabolism at this site. We have recently demonstrated that inhibition of SphK2 induces unrecoverable ER stress leading to apoptosis of multiple myeloma cells and this ER stress-inducing mechanism is most likely also applicable to a range of cell types, including those of ALL, thus impacting on its development in our model [48].

The lower WBC in leukemic MARS2 was interesting and although altered trafficking of lymphoid cells in SphK2^−/−^ animals might be an explanation for this observation, previous reports have demonstrated increased plasma sphingosine-1-phosphate (S1P) and resultant increased lymphocyte mobilization in SphK2^−/−^ mice [49]. All but one MARS2 mouse that did not develop ALL went on to develop solid tumors at a time closer to the previously reported latency (median of 266 days) for solid tumors in ARF deficient animals [31]. Since the tumors that emerged in this study could not be definitively classified, it is not possible to comment on the effects of SphK2 loss on the development of other malignancies.

## Conclusions

We have previously demonstrated the role of SphK2 in ALL driven by BCR/ABL1 and the potential therapeutic application of SphK2 inhibitors in this disease. In this study we demonstrate that SphK2 also plays a role in the development of BCR/ABL1 negative ALL with genetic deletion of SphK2 reducing disease incidence. These findings further support and broaden the potential application of SphK2 inhibitors in the treatment of ALL.

## Additional file

Additional file 1: Additional Data: Table S1, Figures S1-4. (DOCX 6406 kb)

## Abbreviations

ALL: Acute lymphoblastic leukemia
AML: Acute myeloid leukemia
ARF^−/−^: Mice lacking the p19ARF product of the INK4a/ARF locus
ARF^fl/fl^: Mice where the ARF gene had been floxed
ER: Endoplasmic reticulum
MAR: Mx1.Cre.ARF^fl/fl^.Rag1^−/−^
MARS2: Mx1.Cre.ARF^fl/fl^.Rag1^−/−^.SphK2^−/−^
S1P: Sphingosine-1-phosphate
SphK: Sphingosine kinase
