# Supplementary material for: Sphingosine kinase 2 supports the development of BCR/ABL-independent acute lymphoblastic leukemia in mice
Source: Biomark Res. 2018 Feb 5;6:6. doi: 10.1186/s40364-018-0120-4 (PMC5800079; doi:10.1186/s40364-018-0120-4)
Supplement: Additional file 1: — Additional Data: Table S1, Figures S1-4. (DOCX 6406 kb) [file 40364_2018_120_MOESM1_ESM.docx]

**Additional Information**

**Table S1**

| Gene | Forward Primer | Reverse Primer | Product Size (bp) |
| --- | --- | --- | --- |
| ARF | TACTGCAGCCAGACCACTAGG | CGGAGATTGAGAAAGCGG | 263 WT  360 Floxed |
| ARF | TACTGCAGCCAGACCACTAGG | CGAGGAAATGTTGGCGCC | 493 KO |
| Mx1.Cre | GCGGTCTGGCAGTAAAAACTAT | GTGAAACAGCATTGCTGTCACTT | 100 |
| IL2 | CTAGGCCACAGAATTGAAAGATC | GTAGGTGGAAATTCTAGCATCAT | 324 |
| Rag1 | GAGGTTCCGCTACGACTCTG  TGGATGTGGAATGTGTGCGAG | CCGGACAAGTTTTTCATCGT | 474 WT  530 Mut |
| SphK2 | TCTGGAGACGGGCTGCTTTA  CGCTATCAGGACATAGCGTT | GCACCCAGTGTGAATCGAGC | 300 WT  350 KO |


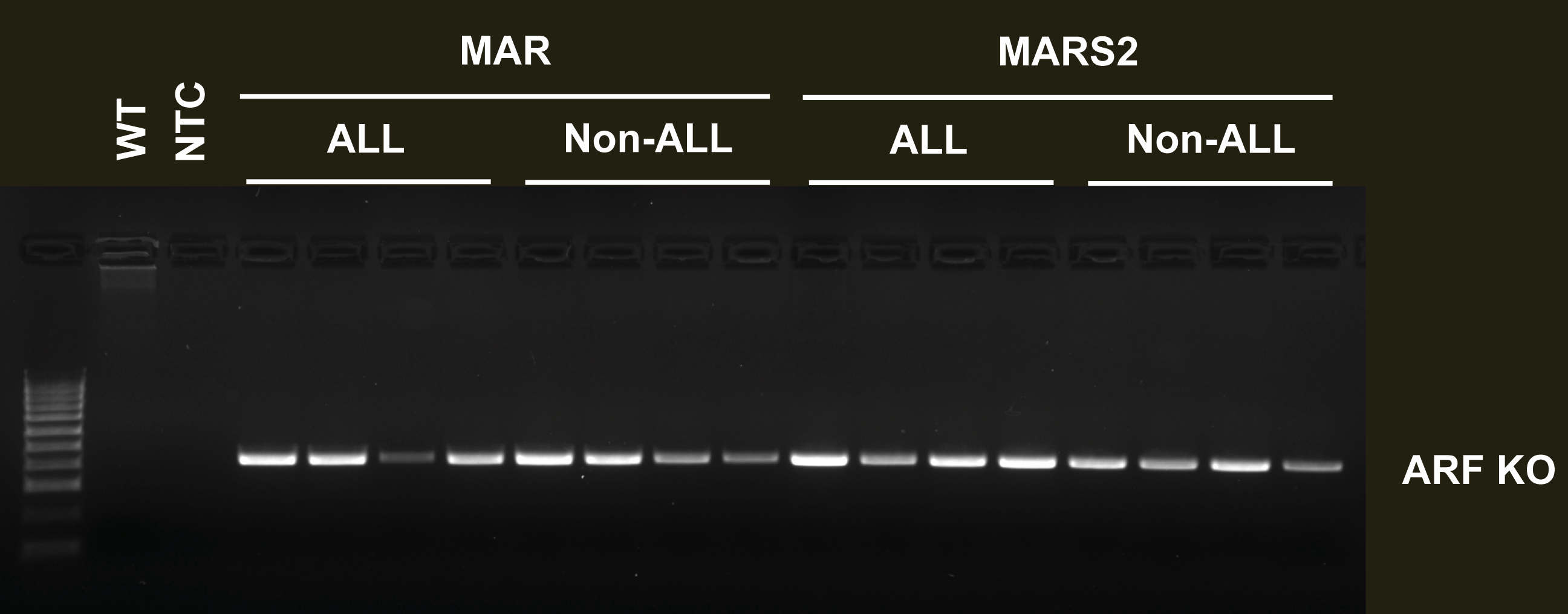


**Figure S1. Verification of deletion of ARF following PolyI:PolyC treatment.** Amplification of ARF in spleen cells recovered from mice at cull using the second set of primers in Table S1. These primers amplify across the region of ARF deleted following polyI:polyC treatment and so do not produce a band unless ARF has been deleted. WT indicates material from a mouse lacking Mx1.Cre. NTC – No template control.


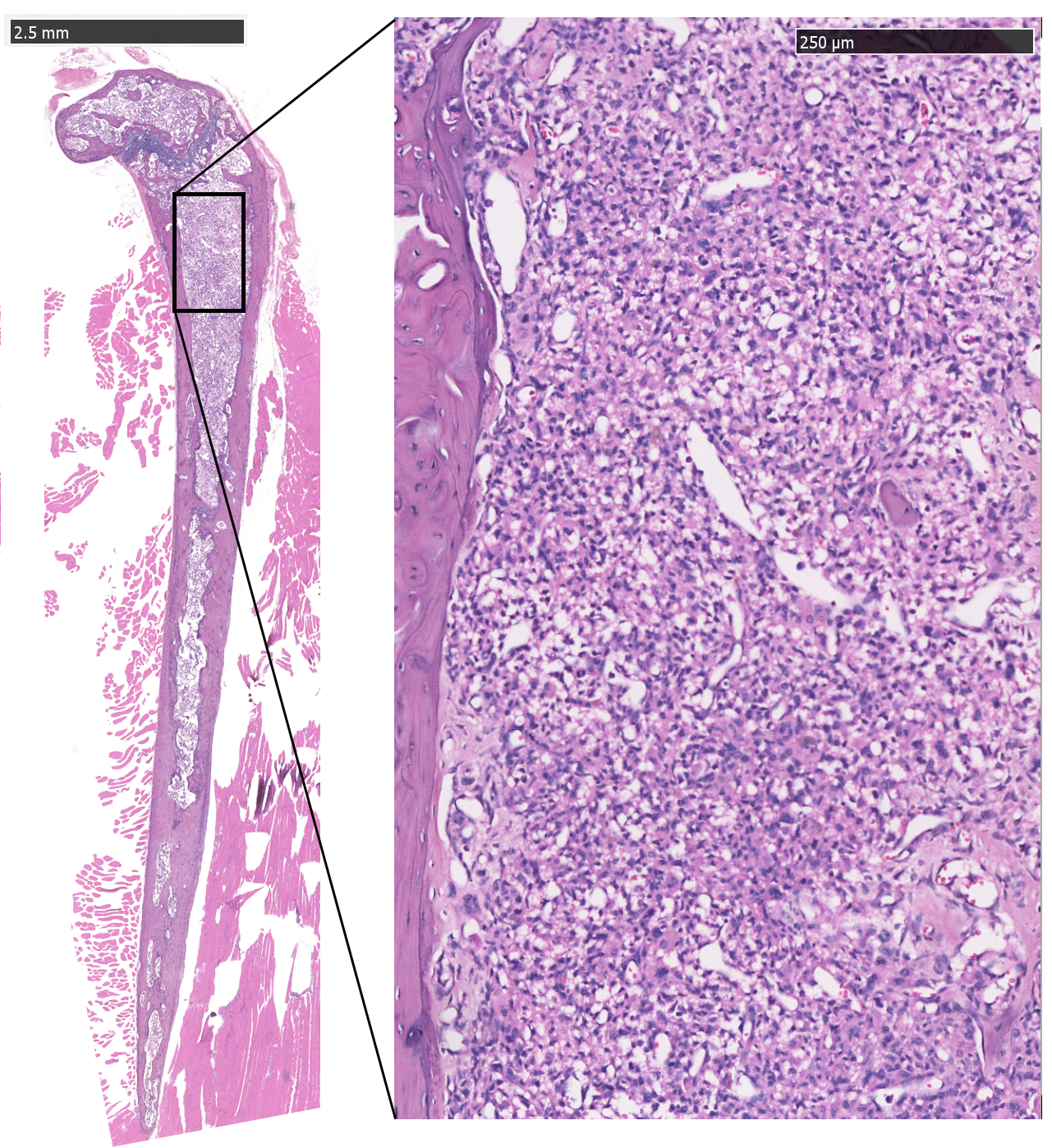


**Figure S2. Example of marrow from a MAR mouse without ALL.** Magnification bars are provided.

**
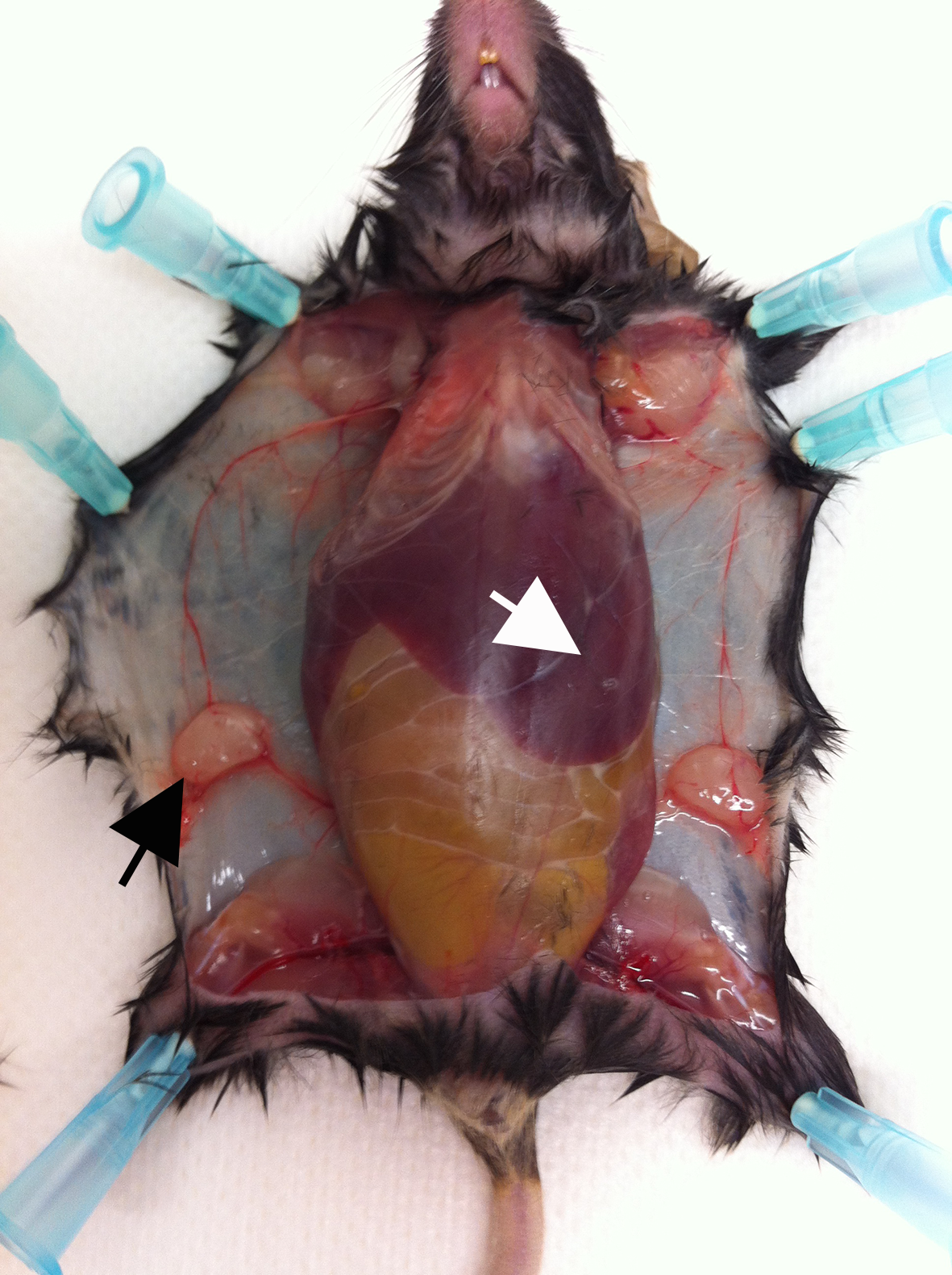
**

**Figure S3. One MAR mouse demonstrated enlarged lymph nodes.** White arrow indicates the spleen and the Black arrow an enlarged lymph node.


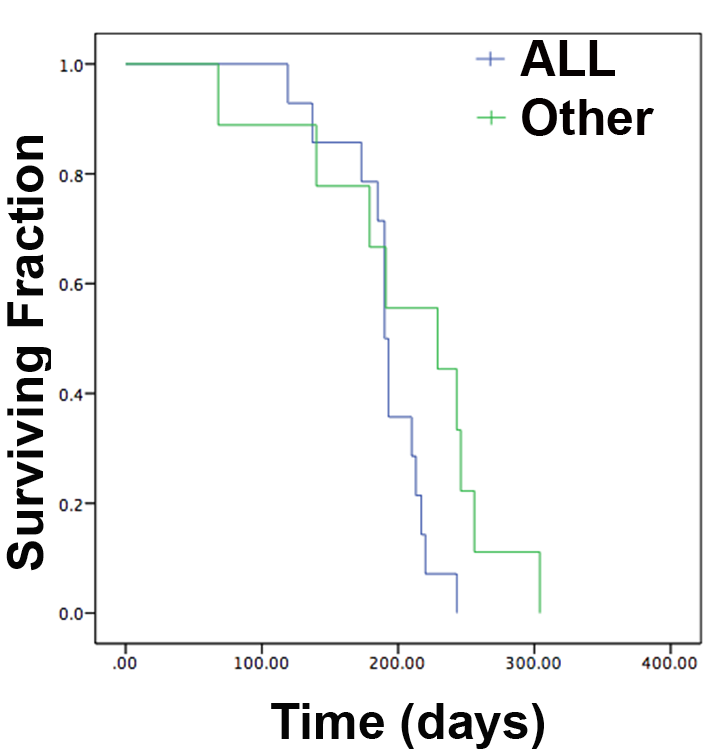


**Figure S4. Comparison of MAR mice death due to ALL and other causes.**
